# Predictive Value of Hyperintense Acute Reperfusion Marker in Transient Ischemic Attacks: Stroke Recurrence and TOAST Classification

**DOI:** 10.3390/brainsci15020170

**Published:** 2025-02-10

**Authors:** Sang-Hoon Han, Kyu-Sun Yum

**Affiliations:** 1 Naval Maritime Medical Center, Changwon 51640, Republic of Korea; hansteven@naver.com; 2 Chungbuk National University College of Medicine, Department of Neurology, Chungbuk National University Hospital, Cheongju 28644, Republic of Korea

**Keywords:** transient ischemic attack, hyperintense acute reperfusion marker, post-contrast fluid-attenuated inversion recovery, Trial of Org 10172 in Acute Stroke Treatment classification, magnetic resonance imaging

## Abstract

Background: Transient ischemic attack (TIA) is a recognized precursor of stroke that also indicates a high risk of recurrence. The Hyperintense Acute Reperfusion Marker (HARM), which is linked to blood–brain barrier disruption, may serve as a novel imaging biomarker for TIA. Methods: A retrospective cohort study of 715 patients with TIA evaluated the predictive value of HARM for stroke recurrence and its association with TOAST subtype. Imaging findings, including those of diffusion-weighted imaging, were analyzed using logistic regression. Results: HARM-positive patients had significantly higher recurrence rates at 3 months (20.9%; odds ratio [OR] = 5.3) and 1 year (30.2%; OR = 5.8) compared to HARM-negative patients, relative to other imaging markers (*p* < 0.001). HARM was significantly associated with the cardioembolic stroke (*p* < 0.001). Conclusions: HARM is a promising imaging biomarker for predicting stroke recurrence and etiology in patients experiencing TIA. Incorporating HARM into clinical frameworks may enhance diagnostic and prognostic accuracy.

## 1. Introduction

Stroke is a leading cause of mortality and long-term disability worldwide, imposing a significant public health and socioeconomic burden [1]. With the rapidly aging global population, the incidences of stroke and other age-related diseases are projected to increase, further straining healthcare systems [2,3]. Understanding stroke mechanisms and identifying individuals at high risk for stroke are essential for effective prevention and management [4].

Transient ischemic attack (TIA) is a critical predictor of stroke, with an incidence rate of 1.19 per 1000 person-years [5]. Nearly 30% of patients who experience TIA develop a stroke within a median follow-up period of 8.86 years [5]. This substantial risk underscores the importance of improving the methods for identifying and managing high-risk patients.

Numerous studies have explored the risk factors of stroke recurrence in patients with TIA. One study identified factors, such as severe symptomatic extra- or intracranial arterial disease, motor weakness, speech impairment, prior TIA, excessive alcohol intake, heart failure, and vertebrobasilar events [6]. Biomarkers have emerged as a promising research area. For example, elevated levels of interleukin-6 and YKL-40 have been linked to an increased risk of stroke recurrence in patients with TIA [7]. These findings highlight the potential role of molecular markers in elucidating pathophysiological mechanisms underlying stroke recurrence. Another widely used tool is the ABCD2 score, which estimates recurrence risk by integrating age, initial blood pressure, clinical presentation, symptom duration, and diabetes status [8]. While this score is useful, one key limitation of the ABCD2 score is that it does not assess vascular integrity or ongoing cerebrovascular pathology, such as arterial stenosis, perfusion deficits, or blood–brain barrier (BBB) dysfunction. This means that in certain populations—particularly those with cardioembolic stroke or other mechanisms where vascular integrity plays a crucial role—it may fail to accurately stratify risk [9]. As a result, imaging studies have played a crucial role, offering valuable insights into the structural and functional changes associated with TIA and its sequelae [10]. Diffusion-weighted imaging (DWI) has been extensively studied and is recognized for its ability to predict stroke recurrence by detecting TIA-related ischemic changes [11,12]. Positive diffusion restriction in patients with TIA had a 9.1% risk of stroke within 1 year compared to 2.1% of DWI-negative patients, highlighting the utility of DWI in predicting stroke recurrence risk [13]. Additionally, DWI-positive lesions help predict stroke etiology: single lesions are strongly associated with small-vessel occlusion (SVO), whereas multiple lesions are more commonly associated with large-artery diseases and cardioembolic (CE) sources [14]. While DWI is highly effective in detecting acute infarcts, it does not assess broader aspects of vascular pathology. BBB disruption, cerebral perfusion abnormalities, and ongoing vascular remodeling play a crucial role in stroke progression and recurrence risk [15,16,17]. Additionally, various imaging techniques, including DWI, perfusion-weighted imaging (PWI), fluid-attenuated inversion recovery (FLAIR), and gradient echo imaging (GRE), have been investigated for their relevance in understanding stroke mechanisms [18].

Within this framework, the Hyperintense Acute Reperfusion Marker (HARM) has emerged as a key imaging biomarker that provides complementary insights into stroke risk stratification. HARM is characterized by delayed hyperintensity of the cerebrospinal fluid in FLAIR magnetic resonance imaging (MRI) sequences, reflecting blood–brain barrier (BBB) disruption [19]. This imaging marker has been observed in various patient populations, including those with intracerebral hemorrhage [20], multiple sclerosis [21], acute stroke [22], and TIA [23]. In the context of stroke, BBB disruption has been predominantly observed in cardioembolic (CE) stroke [24]. Unlike DWI, which primarily identifies acute ischemic lesions [25], HARM provides a distinct and complementary perspective by reflecting BBB integrity [20]. While DWI has been established as a crucial tool for detecting infarcted areas and predicting short-term stroke recurrence [4], it does not assess the functional status of the BBB, which plays a key role in long-term cerebrovascular stability [16]. Additionally, the ABCD2 score, though widely used, does not incorporate vascular imaging markers and may underestimate risk in patients with CE stroke, where embolic events and BBB disruption are key factors. By identifying BBB dysfunction, HARM offers an alternative pathway to predict stroke recurrence, particularly in embolic stroke mechanisms, and may enhance risk stratification beyond ABCD2 and DWI. In a study analyzing the relationship between HARM signs and stroke etiology, higher rates of HARM positivity were observed in patients with large artery atherosclerosis (LAA) and CE stroke, whereas its occurrence was significantly lower in patients with SVO [26].

Although HARM has been extensively studied in acute stroke [26,27,28], its clinical utility, particularly in TIA patients, remains underexplored. This study aimed to evaluate whether HARM could serve as a useful biomarker for predicting stroke recurrence and the pathophysiological mechanisms in patients with TIA. Specifically, we seek to compare the predictive value of HARM with established imaging markers, such as DWI, and investigate its relationship with TOAST classifications, thereby offering new insights into the management of patients with TIA.

## 2. Materials and Methods

### 2.1. Ethical Approval and Study Design

This study was approved by the Institutional Review Boards (IRBs) of Chungbuk National University Hospital (CBNUH) (IRB No: 2024-11-007). All methods were conducted in accordance with the CBNUH guidelines and regulations.

### 2.2. Study Population and Patient Selection

This retrospective, single-center study utilized data from an institutional stroke database. Patients aged 18 years or older, admitted with a diagnosis of acute TIA between 7 January 2008, and 31 December 2014, were included. All participants were followed up for one year after admission to monitor outcomes. Among the total cohort, 715 patients met the inclusion criteria. Patients who did not undergo MRI during hospitalization were excluded from the analysis.

### 2.3. Clinical Assessment

All patients were evaluated by an experienced neurologist. The ABCD2 score was determined at the initial visit through patient interviews and physical examination. Medical histories and demographic data were collected at the time of admission. Stroke etiology was classified using the TOAST criteria based on findings from brain imaging, echocardiography, electrocardiography, and laboratory tests conducted during hospitalization. Data on stroke recurrence at 7 days, 3 months, and 1 year were obtained from the Institutional Stroke Registry.

### 2.4. MRI Acquisition

Patients suspected of having acute stroke underwent MRI using standardized protocols with 1.5-T or 3-T scanners (Intera Achieva; Philips, Best, The Netherlands). Imaging sequences included DWI, gradient-echo imaging, time-of-flight MR angiography (MRA) of the intracranial and neck vessels, perfusion-weighted imaging (PWI), post-contrast T1-weighted imaging (T1WI), and post-contrast FLAIR imaging. DWI, MRA, and PWI were prioritized to assess eligibility for intra-arterial thrombolysis. DWI was captured in the axial plane with TR/TE = 3800–5000/45–60 ms, and gradient-echo with TR/TE = 690–1100/15–24 ms. Both intracranial and neck MRAs were acquired with TR/TE = 20–25/3–7 ms. For PWI, Gadovist contrast was administered at 0.2 mM/kg at 4 mL/s, with a 20 mL saline flush, totaling 15 mL of contrast. Echo-planar images were acquired with TR/TE = 1500/30–50 ms. T1-weighted images were obtained using a spin-echo sequence with TR/TE = 400–600/9–12 ms, and FLAIR images were obtained using a turbo-spin-echo sequence with TR/TE = 11000/120–140 ms, flip angle of 90°, and slice thickness of 5 mm. Post-contrast FLAIR imaging was performed 5 min after contrast injection to assess brain lesions and edema.

### 2.5. Image Analysis

Neuroimaging was performed by a neuroradiologist using MRA angiography and perfusion CT. Analysis was conducted on images obtained upon arrival at the emergency department. The evaluation included the presence of acute infarction on DWI (that is, DWI), HARM sign on postcontrast FLAIR imaging, steno-occlusive lesions in the intracranial and extracranial arteries (that is, Stenosis), and perfusion abnormalities on perfusion CT (that is, Perfusion).

**Figure 1:**
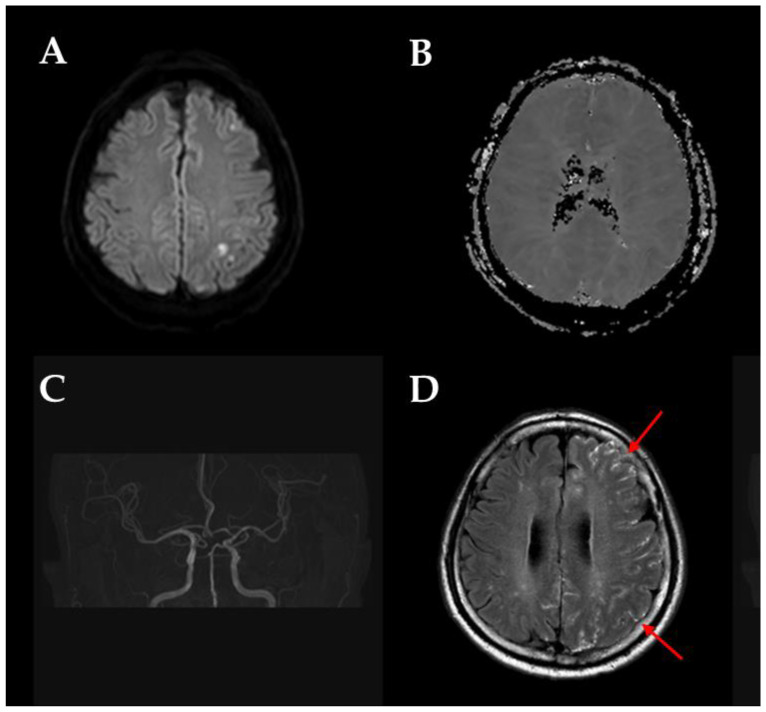
A patient presenting with acute-onset right-sided weakness. (**A**,**B**) DWI and apparent diffusion coefficient (ADC) images show an acute infarction in the left parietal lobe. (**C**) Intracranial time-of-flight angiography reveals no evidence of stenosis or occlusion. (**D**) Post-contrast FLAIR image demonstrates diffuse sulcal enhancement (Hyperintense Acute Reperfusion Marker sign) in the left cerebral sulci (arrows).

HARM was assessed using FLAIR MRI sequences obtained within 24 h after symptom onset. HARM was defined as a delayed hyperintense signal in the cerebrospinal fluid (CSF) on post-contrast FLAIR imaging, which is indicative of BBB disruption. To illustrate the evaluation process, a representative example of a HARM-positive case is provided in Figure 1, demonstrating hyperintense CSF signals on postcontrast FLAIR imaging.

### 2.6. Statistical Analysis

Categorical variables were analyzed using the chi-square test, continuous variables were compared using the independent *t*-test, and non-normally distributed variables were analyzed using the Mann–Whitney U test. Associations between the four imaging markers and stroke recurrence at 7 days, 3 months, and 1 year were assessed using the chi-square test. Multivariate logistic regression analysis was used to evaluate the association between the four imaging markers (HARM, DWI, Stenosis, Perfusion) and stroke recurrence at 7 days, 3 months, and 1 year. The associations between the four imaging markers (HARM, DWI, Stenosis, Perfusion) and TOAST subtypes were assessed using the chi-square test and Fisher’s Exact Test. The dependent variables for each time point were modeled as a function of independent variables, including imaging markers (HARM, DWI, Stenosis, Perfusion) and potential confounders (age, sex, hypertension, diabetes, dyslipidemia, atrial fibrillation, smoking history, previous stroke history, and ABCD2 score) (Table 1). After developing each logistic regression model, the predicted probability of stroke recurrence was calculated for each observation within the dataset. The predicted probabilities were then used to generate receiver operating characteristic (ROC) curves. The area under the curve (AUC) for each ROC curve was calculated to quantify model performance.

All statistical analyses were performed using STATA software (STATA SE 18, StataCorp LLC, College Station, TX, USA), with *p*-values < 0.05 considered statistically significant.

## 3. Results

### 3.1. Patient Demographics

The baseline characteristics of TIA patients with and without HARM are summarized in Table 1. The mean age of the cohort was 63.31 years, with HARM-positive patients being slightly older on average (66.95 years) than HARM-negative patients (63.08 years; *p* = 0.054). Males accounted for 57.8% of the cohort, with a higher proportion in the HARM-positive group (65.1%) than in the HARM-negative group (57.3%; *p* = 0.396).

Among the clinical risk factors, hypertension was present in 64.5% of the patients, while diabetes, dyslipidemia, atrial fibrillation, and smoking were similarly distributed across both groups. High-risk CE sources were more frequent in the HARM-positive patients (25.6%) than in the HARM-negative patients (12.4%; *p* = 0.013). Medium- to low-risk CE sources were also more common in the HARM-positive group (*p* < 0.001). The ABCD2 score, which assesses short-term stroke risk, did not differ significantly between groups.

### 3.2. Stroke Recurrence Rates and Imaging Markers

Table 2 shows the association between the imaging findings and stroke recurrence. At 7 days, HARM (odds ratio [OR] = 3.80, *p* = 0.0302), DWI (OR = 24.57, *p* < 0.0001), Stenosis (OR = 5.07, *p* = 0.0021), and Perfusion (OR = 15.53, *p* < 0.0001) were all significantly associated with a higher recurrence risk. At 3 months, HARM (OR = 5.29, *p* < 0.0001), DWI (OR = 3.61, *p* = 0.0001), Stenosis (OR = 2.43, *p* = 0.0054), and Perfusion (OR = 4.27, *p* = 0.0012) remained significant predictors of recurrence. At 1 year, HARM (OR = 5.76, *p* < 0.0001), DWI (OR = 2.34, *p* = 0.0015), Stenosis (OR = 2.29, *p* = 0.0019), and Perfusion (OR = 3.33, *p* = 0.0049) were significantly associated with stroke recurrence.

### 3.3. Multivariable Logistic Regression Analysis of Stroke Recurrence Prediction by Imaging Findings

Table 3 presents the results of the multivariate logistic regression analysis for stroke recurrence at three time points: 7 days, 3 months, and 1 year. The analysis evaluated the relationship between the four imaging markers—HARM, DWI, Stenosis, and Perfusion—and the risk of stroke recurrence at each time point, adjusting for various confounding factors. At day 7, HARM was not significantly associated with stroke recurrence (OR = −0.070, 95% CI: −1.842–1.702, *p* = 0.938). However, DWI was significantly associated with an increased risk, with an OR of 2.684 (95% CI: 0.587–4.781, *p* = 0.012). Stenosis showed a borderline significant effect (OR = 1.218, 95% CI: −0.042 to 2.478, *p* = 0.058), while perfusion was significantly associated with an increased risk of stroke recurrence (OR = 2.127, 95% CI: 0.724 to 3.530, *p* = 0.003). At 3 months, HARM remained a significant predictor of stroke recurrence (OR = 1.460, 95% CI: 0.460–2.460, *p* = 0.004), similar to DWI (OR = 1.232, 95% CI: 0.498–1.966, *p* = 0.001). Stenosis had a significant but moderate effect on recurrence risk (OR = 0.845, 95% CI: 0.145–1.545, *p* = 0.018), whereas Perfusion did not show any significant effect (OR = 0.482, 95% CI: −0.666 to 1.629, *p* = 0.411). At 1 year, HARM was again a significant predictor of stroke recurrence (OR = 1.769, 95% CI: 0.923–2.616, *p* < 0.001). Interestingly, DWI showed a decrease in recurrence risk (OR = 0.698, 95% CI: 0.108–1.288, *p* = 0.020), while stenosis was significantly associated with a reduced risk of recurrence (OR = 0.868, 95% CI: 0.287–1.448, *p* = 0.003). Perfusion did not show any significant association with stroke recurrence at 1 year (OR = 0.081, 95% CI: −0.975 to 1.137, *p* = 0.880).

### 3.4. Model Performance in Predicting Stroke Recurrence at 7 Days, 3 Months, and 1 Year

**Figure 2:**
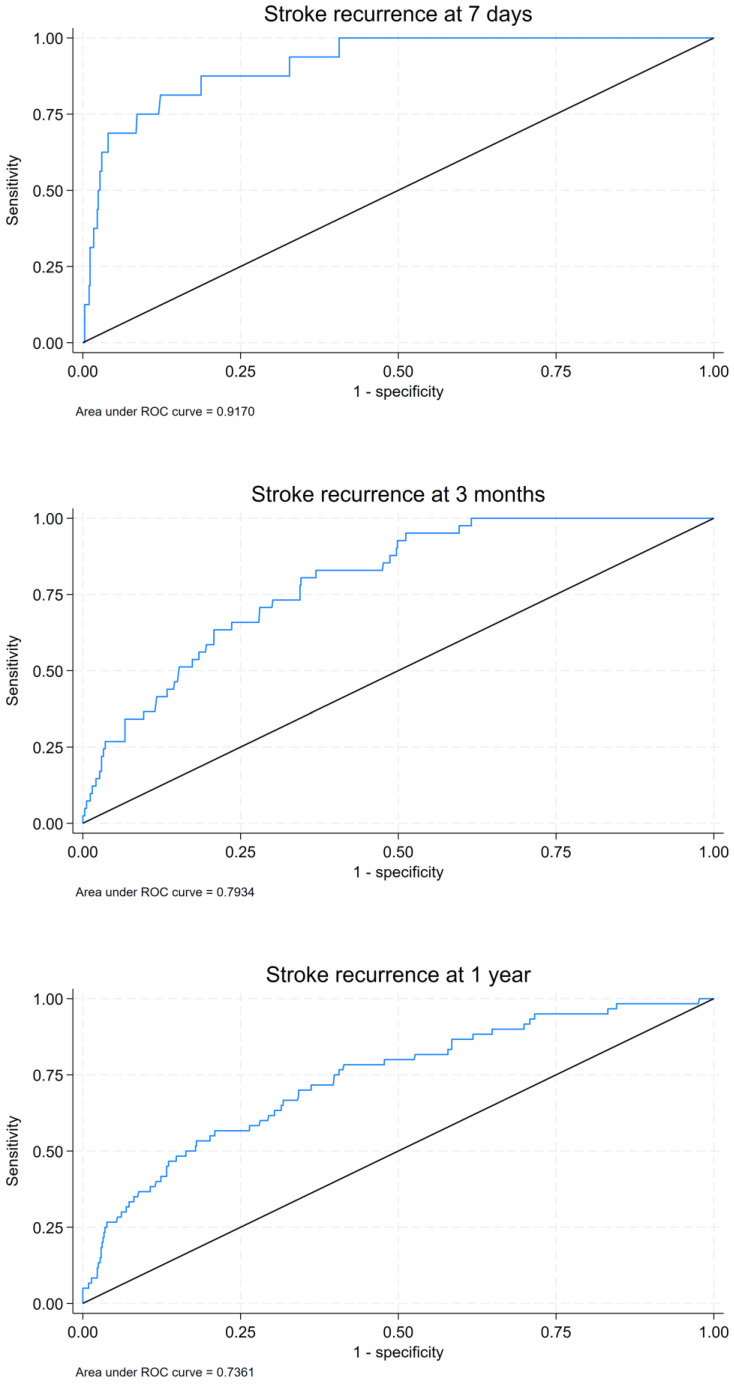
Receiver operating characteristic (ROC) curve for stroke recurrence at 7 days, 3 months, and 1 year. The blue line represents stroke recurrence, and the black line indicates the reference (AUC = 0.5).

Logistic regression models were developed to predict stroke recurrence at 7 days, 3 months, and 1 year, which were evaluated using receiver operating characteristic (ROC) curves (Figure 2). The 7-day stroke recurrence prediction model had an AUC of 0.9170, demonstrating excellent performance. The 3-month and 1-year models also showed AUC values of 0.7934 and 0.7361, respectively.

### 3.5. Correlation of HARM Sign with TOAST Subtypes

The analysis revealed a significant association between the HARM sign and TOAST subtypes, as demonstrated by chi-square tests (Table 4) and logistic regression analysis (Table 5 and Table 6). Specifically, a statistically significant association was observed between HARM findings and TOAST subtypes (χ^2^(5) = 42.40, *p* < 0.001). HARM positivity was significantly associated with CE etiology (χ^2^(1) = 35.78, *p* < 0.001) and undetermined negative (UDN) etiology (χ^2^(1) = 14.38, *p* < 0.001). Other etiologies, including LAA, SVO, stroke of other determined etiology (OD), and stroke of undetermined etiology with two or more causes (UD2), did not show a statistically significant association with the HARM sign. Table 5 and Table 6 present the results of multivariate logistic regression analyses examining the associations of the four imaging markers (HARM, DWI, Stenosis, Perfusion) with the CE and UDN subtypes. These findings demonstrate that the presence of the HARM sign serves as an independent predictive factor for both CE and UDN subtypes, exerting its influence regardless of the presence of other imaging markers.

## 4. Discussion

This study suggests that the HARM sign may be a valuable biomarker for predicting stroke recurrence and for understanding the underlying pathophysiological mechanisms in patients with TIA. Our findings showed that HARM is significantly associated with long-term stroke recurrence at both 3 months and 1 year. Additionally, HARM was strongly correlated with the CE stroke subtype of the TOAST classification, further supporting its potential role in identifying stroke pathophysiology. While DWI demonstrated the most significant predictive value for 7-day recurrence, HARM appeared to play a prominent role in long-term predictions. These results suggest that incorporating HARM into the diagnostic and prognostic framework for TIA may help improve predictive accuracy, complementing other well-established imaging markers such as DWI.

Brain imaging findings are widely recognized as predictors of stroke mechanisms and recurrence in TIA diagnosis, with DWI being a well-established representative marker [29,30]. However, early DWI may fail to detect hyperintense lesions if they are absent or too small to identify [31]. Additionally, in cases of TIA caused by reduced cerebral perfusion, a diagnosis based solely on the presence or absence of lesions on DWI can be challenging [32]. To address these limitations, vascular and perfusion imaging in addition to DWI can be used for diagnosis [33].

The HARM sign is an imaging marker of BBB disruption in patients with ischemic stroke [19]. With the increasing use of contrast-enhanced MRI in acute stroke imaging, the HARM sign is being observed more frequently, including in TIA cases [22,23]. In ischemic stroke, HARM is associated with embolic stroke mechanisms, particularly in patients with LAA or CE [26]. However, the significance of the HARM sign in patients with TIA has not yet been clearly established.

In TIA, stroke recurrence is commonly predicted using the ABCD2 score, and a positive DWI finding is recognized as valuable predictive marker [11]. In our study, the recurrence rates at 7 days, 3 months, and 1 year were 2.24%, 5.73%, and 8.4%, respectively. Patients with positive DWI findings showed recurrence rates of 5.4% within 7 days, 10% within 3 months, and 12.5% within 1 year, which are higher than those reported in the TIAregistry.org project but consistent with other studies [32,34,35]. These findings are consistent with those of previous studies, suggesting that DWI-positive findings are associated with higher recurrence rates. This is likely due to variations between the data published in 2016 and older studies, potentially driven by the more rapid implementation of newer and more effective secondary prevention strategies. As our study population included patients enrolled before 2014, it is likely that studies involving more recent cohorts have reported lower recurrence rates.

Similar to DWI, HARM was associated with higher recurrence rates when positive, with rates of 7.0% at 7 days, 20.9% at 3 months, and 30.2% at 1 year. However, while DWI demonstrated strong predictive power for early stroke recurrence (particularly at 7 days), HARM showed increasing predictive significance over longer time frames (3 months and 1 year). This suggests that HARM captures distinct pathophysiological mechanisms related to stroke recurrence, likely involving ongoing BBB disruption and delayed reperfusion injury, which are not detected by DWI alone. Furthermore, given that ABCD2 primarily relies on clinical parameters without incorporating vascular imaging, the combination of HARM with ABCD2 may improve long-term risk stratification, especially in patients with CE stroke. Future studies should explore the integration of HARM into standardized risk prediction models to optimize treatment strategies.

Consistent with these findings, the results of our multivariate logistic regression analysis further demonstrated the complementary roles of DWI and HARM in stroke risk assessment. In particular, DWI demonstrated strong predictive power for recurrence at 7 days and 3 months, whereas HARM emerged as a significant predictor of recurrence at 3 months and 1 year. Stenosis and Perfusion had significant effects at specific time points (mainly at 3 months and 1 year); however, Perfusion did not show a significant impact at 7 days and 1 year. These findings suggest that HARM could be a useful marker for predicting long-term recurrence, whereas DWI plays a particularly important role in predicting early recurrence. Additionally, the effects of Stenosis and Perfusion varied over time, indicating that the role of each factor in predicting recurrence risk may change over time.

The 7-day stroke recurrence prediction model had an AUC of 0.9170, demonstrating excellent performance. The 3-month and 1-year models also showed AUC values of 0.7934 and 0.7361, respectively, indicating good predictive performance. The differences in AUC values based on the inclusion of HARM at each time point reveal that for 7-day recurrence prediction, there was little difference between the models with and without HARM (AUC = 0.9170 vs. 0.9166). However, for the 3-month and 1-year recurrence predictions, the model including HARM showed improved performance, with AUC values of 0.7934 and 0.7361, respectively, compared with 0.7504 and 0.6770, respectively, for the models without HARM. These results suggest that while HARM does not significantly impact short-term (7-day) recurrence prediction, it plays a valuable role in enhancing the accuracy of long-term (3-month and 1-year) recurrence predictions.

The comparison between models with and without HARM demonstrated that including HARM improved prediction accuracy, particularly for long-term recurrence, with HARM-inclusive models yielding higher AUC values than models without HARM. This study highlights the potential for incorporating HARM into existing predictive models to improve the prognostic accuracy for TIA patients with TIA.

Based on the results of this study alone, it cannot be concluded that HARM is a stronger predictor than other imaging markers. However, HARM appears to be a meaningful predictor of stroke recurrence in patients with TIA. Incorporating the HARM sign alongside established clinical measures, such as the ABCD2 score, and imaging biomarkers, such as DWI, may enhance predictive accuracy.

HARM showed a strong association with CE stroke in the TOAST classification. This is likely because HARM reflects pathophysiological processes related to blood–brain barrier disruption and reperfusion injury. These findings suggest that HARM can provide new insights into the existing TOAST classification framework. Previous studies on ischemic stroke reported that HARM is strongly associated with embolic stroke. Our previous study demonstrated similar findings [26]. The chi-square test results showed that the HARM sign was significantly associated with TIA mechanisms, particularly CE and UDN. HARM is caused by BBB disruption following stroke and has been linked to reperfusion injury in previous studies [19]. CE strokes have higher rates of spontaneous reperfusion than other mechanisms, which may explain the higher prevalence of HARM in patients with CE TIA [36,37]. In multivariate logistic regression, the HARM sign showed a significant positive correlation with CE, suggesting that it could independently serve as a predictive marker for CE. The HARM sign also showed a significant negative association in the UDN group. Its stronger association with CE than with other imaging markers suggests that the HARM sign may provide insight into the mechanisms underlying strokes classified as embolic strokes of undetermined source (ESUS). Further studies investigating the relationship between HARM and stroke mechanisms in patients with ESUS are required to confirm this hypothesis.

These findings suggest that the presence of HARM in TIA patients may have clinical implications in therapeutic decision making. Given its strong association with CE stroke, identifying HARM positivity could aid in determining whether anticoagulation therapy is more appropriate than antiplatelet therapy in secondary stroke prevention. Furthermore, in cases of ESUS, HARM detection may serve as an indicator for further diagnostic evaluation, such as the use of an implantable loop recorder (ILR) to detect occult atrial fibrillation. By integrating HARM into the clinical workflow, physicians may be able to refine treatment strategies and enhance individualized patient management.

## 5. Limitations

This study has some limitations. First, as this was a retrospective study conducted at a single center, selection bias may have been introduced. The high stroke recurrence rate observed in the present study may reflect the timing of stroke occurrence in the study population [34]. Second, despite its promising predictive value, HARM detection is highly dependent on imaging protocols, including contrast agent timing, FLAIR sequence acquisition, and scanner settings, which may vary across institutions. These variations could influence the reported prevalence of HARM and its prognostic accuracy. Additionally, the lack of standardized acquisition and interpretation criteria for HARM may limit its clinical applicability. Future research should focus on developing consensus guidelines for HARM imaging and interpretation to ensure reproducibility across different centers and patient populations. Third, the imaging timing varied, which may have influenced the detection rates of both DWI and HARM. Our study included patients with symptom onsets ranging from the hyperacute phase (within 6 h) to the acute phase (24 h to 1 week), and this variability was not controlled for. DWI lesions may not be observed or may disappear depending on the timing of imaging, which is presumed to have an impact on the findings [25]. Fourth, the patient treatment methods were not standardized, potentially affecting stroke recurrence rates and imaging marker occurrence. For example, the analysis did not account for the rate of anticoagulation therapy or administration of intravenous thrombolysis in some patients, which could have affected the results. Finally, the number of patients with HARM was limited. Although statistically significant associations were observed, the small sample size limits the generalizability of the findings. Conducting studies with larger cohorts could help address this limitation. Future multicenter studies are needed to validate the utility of HARM in larger patient populations. Additionally, developing methods to integrate HARM with the ABCD2 score and DWI as a clinical tool will be essential for evaluating its applicability in real-world clinical settings.

## 6. Conclusions

This study suggests that HARM could serve as a useful biomarker for predicting stroke recurrence and understanding the pathophysiological mechanisms in patients with TIA. Specifically, HARM may complement existing tools, such as DWI and ABCD2 scores, in predicting long-term recurrence. Its association with CE stroke and specific TOAST classifications indicate its potential to provide additional insights into stroke pathophysiology. These findings highlight the possibility that incorporating HARM into established clinical and imaging frameworks could enhance predictive accuracy and support more tailored management strategies for patients with TIA.

Beyond risk stratification, HARM positivity may also inform therapeutic decision making, particularly in determining whether anticoagulation therapy is warranted for secondary stroke prevention. Additionally, its presence in ESUS cases could prompt further diagnostic evaluation to identify underlying embolic sources. These findings suggest that incorporating HARM into clinical workflows could enhance individualized patient management.

Further multicenter studies are necessary to confirm the utility of HARM in larger patient populations. Additionally, efforts to integrate HARM with the ABCD2 score and DWI into practical clinical tools will be essential to fully evaluate its applicability in real-world settings. Such research could help improve the outcomes for patients with TIA and contribute to stroke prevention strategies.

## Figures and Tables

**Table 1:** Baseline characteristics of patients with transient ischemic attack (TIA) and their comparisons by Hyperintense Acute Reperfusion Marker (HARM) sign.

Characteristics	Total (*n* = 715)	HARM-Positive (*n* = 43)	HARM-Negative (*n* =672)	*p*-Value
Age	63.31 ± 13.260	66.95 ± 12.436	63.08 ± 13.286	0.057
Male	413 (57.8%)	28 (65.1%)	385 (57.3%)	0.396
Risk factor				
Hypertension	461 (64.5%)	22 (51.2%)	439 (65.3%)	0.086
Diabetes mellitus	153 (21.4%)	8 (18.6%)	145 (21.6%)	0.788
Dyslipidemia	235 (32.9%)	11 (25.6%)	224 (33.3%)	0.378
Atrial fibrillation	74 (10.3%)	6 (14.0%)	68 (10.1%)	0.587
Smoking	278 (38.9%)	18 (41.9%)	260 (38.7%)	0.801
History of stroke	137 (19.2%)	9 (20.9%)	128 (19.0%)	0.916
Prior TIA before index TIA	85 (11.9%)	4 (9.3%)	81 (12.1%)	0.766
Laboratory finding				
SBP, initial	149.88 ± 25.086	154.84 ± 28.175	149.56 ± 24.865	0.318
DBP, initial	82.74 ± 14.846	85.16 ± 13.445	82.59 ± 14.927	0.324
Total cholesterol	171.49 ± 39.029	178.19 ± 38.777	171.06 ± 39.035	0.246
Cardioembolic source				
High-risk	94 (13.1%)	11 (25.6%)	83 (12.4%)	0.013 *
Medium/low-risk	55 (7.7%)	13 (30.2%)	42 (6.3%)	<0.001 *
ABCD2 score	2.74 ± 1.77	2.58 ± 1.88	2.75 ± 1.77	0.456

ABCD2, age, initial blood pressure, clinical presentation, symptom duration, and diabetes status; SBP systolic blood pressure; DBP diastolic blood pressure. * indicates statistical significance (*p* < 0.05).

**Table 2:** Comparison of stroke recurrence rates in patients with TIA.

Variable	Positive	Negative	OR	*p*-Value
HARM sign				
At 7 days	3 (7.0%)	13 (1.9%)	3.802 (1.041–13.885)	0.03
At 3 months	9 (20.9%)	32 (4.8%)	5.294 (2.341–11.972)	<0.001
At 1 year	13 (30.2%)	47 (7.0%)	5.762 (2.819–11.781)	<0.001
DWI				
At 7 days	15 (5.4%)	1 (0.2%)	24.566 (3.226–187.048)	<0.001
At 3 months	28 (10%)	13 (3.0%)	3.607 (1.835–7.091)	<0.001
At 1 year	35 (12.5%)	25 (5.7%)	2.343 (1.369–4.009)	0.001
Arterial stenosis				
At 7 days	12 (4.4%)	4 (0.9%)	5.065 (1.617–15.869)	0.002
At 3 months	24 (8.8%)	17 (3.8%)	2.425 (1.278–4.602)	0.005
At 1 year	34 (12.5%)	26 (5.9%)	2.291 (1.342–3.912)	0.002
Perfusion abnormality				
At 7 days	6 (18.8%)	10 (1.5%)	15.530 (4.252–51.061)	<0.001
At 3 months	6 (18.8%)	35 (5.1%)	4.273 (1.651–11.054)	0.001
At 1 year	7 (21.9%)	53 (7.8%)	3.328 (1.375–8.054)	0.005

OR, odds ratio.

**Table 3:** Multivariable Logistic regression for stroke recurrence periods.

Stroke Recurrence Period	OR	*p*-Value
7 day		
HARM	−0.070 (−1.842–1.702)	0.938
DWI	2.684 (0.587–4.781)	0.012
Stenosis	1.218 (−0.042–2.478)	0.058
Perfusion	2.127 (0.724–3.530)	0.003
3 month		
HARM	1.460 (0.460–2.460)	0.004
DWI	1.232 (0.498–1.966)	0.001
Stenosis	0.845 (0.145–1.545)	0.018
Perfusion	0.482 (−0.666–1.629)	0.411
1 year		
HARM	1.769 (0.923–2.616)	<0.001
DWI	0.698 (0.108–1.288)	0.020
Stenosis	0.868 (0.287–1.448)	0.003
Perfusion	0.081 (−0.975–1.137)	0.880

**Table 4:** Correlation between HARM sign and TOAST classification.

HARM	TOAST Classification						
	1	2	3	4	5	6	Total
Negative	159	68	87	16	1	341	672
Positive	9	2	20	3	0	9	43
Total	168	70	107	19	1	350	715
Odds ratio	0.1676	1.3681	35.7796	3.299	0.0641	14.3753	42.4022
*p*-value	0.682	0.242	<0.001 *	0.069	0.800	<0.001 *	<0.001 *

1 Large artery atherosclerosis. 2 Small vessel occlusion. 3 Cardioembolism. 4 Stroke of other determined etiology. 5 Stroke of undetermined etiology, two or more causes 6 Stroke of undetermined etiology, negative. * indicates statistical significance (*p* < 0.05).

**Table 5:** Logistic regression analysis between imaging markers and cardioembolism.

Logistic Regression				
	*p*-Value	Odds Ratio	CI	
HARM	<0.001 *	4.975	2.427	10.195
DWI	<0.001 *	3.133	2.004	4.898
Stenosis	0.012 *	0.539	0.334	0.872
Perfusion	0.505	1.371	0.542	3.466

* indicates statistical significance (*p* < 0.05).

**Table 6:** Logistic regression analysis between imaging markers and undetermined-negative etiology.

Logistic Regression				
	*p*-Value	Odds Ratio	CI	
HARM	<0.001 *	0.176	0.073	0.421
DWI	<0.001 *	0.014	0.095	0.205
Stenosis	<0.001 *	0.030	0.104	0.226
Perfusion	0.122	0.346	0.091	1.326

* indicates statistical significance (*p* < 0.05).

## Data Availability

This study utilized data from the institutional stroke registry, which contains personal information requiring anonymization. Upon reasonable request, the data can be provided by the corresponding author after anonymization.

## Abbreviations

The following abbreviations are used in this manuscript:

ABCD2	age, initial blood pressure, clinical presentation, symptom duration, and diabetes status
AUC	area under the curve
BBB	blood–brain barrier
CE	cardioembolism/cardioembolic
CI	confidence interval
DWI	diffusion-weighted imaging
ESUS	embolic stroke of undetermined source
FLAIR	fluid-attenuated inversion recovery
GRE	gradient echo imaging
HARM	hyperintense acute reperfusion marker
IV	intravenous
LAA	large artery atherosclerosis
MRI	magnetic resonance imaging
OR	odds ratio
PWI	perfusion-weighted imaging
ROC	receiver operating characteristic
SVO	small-vessel occlusion
TIA	transient ischemic attack
TOAST	Trial of ORG 10172 in Acute Stroke Treatment
UDN	Undetermined-negative
UD2	Stroke of Undetermined Etiology with Two or More Causes
